# Interleukin-6: An Under-Appreciated Inducer of Thermogenic Adipocyte Differentiation

**DOI:** 10.3390/ijms25052810

**Published:** 2024-02-28

**Authors:** Ádám Radványi, Tamás Röszer

**Affiliations:** Department of Pediatrics, Faculty of Medicine, University of Debrecen, H-4032 Debrecen, Hungary; radvanyi.adam@med.unideb.hu

**Keywords:** obesity, thermogenesis, adipocyte, inflammation

## Abstract

Adipose tissue inflammation is a key factor leading to obesity-associated immune disorders, such as insulin resistance, beta cell loss in the pancreatic islets, meta-inflammation, and autoimmunity. Inhibiting adipose tissue inflammation is considered a straightforward approach to abrogate these diseases. However, recent findings show that certain pro-inflammatory cytokines are essential for the proper differentiation and functioning of adipocytes. Lipolysis is stimulated, and the thermogenic competence of adipocytes is unlocked by interleukin-6 (IL-6), a cytokine that was initially recognized as a key trigger of adipose tissue inflammation. Coherently, signal transducer and activator of transcription 3 (STAT3), which is a signal transducer for IL-6, is necessary for thermogenic adipocyte development. Given the impact of thermogenic adipocytes in increasing energy expenditure and reducing body adiposity, functions of IL-6 in the adipose tissue have gained attention recently. In this review, we show that IL-6 signaling may protect from excess fat accumulation by stimulating thermogenesis in adipocytes.

## 1. The Role of Inflammation in Adipose Tissue Functioning

Adipose tissue is a key metabolic organ that determines energy expenditure and the long-term maintenance of body weight. Adipocytes in the adipose tissue serve as energy reserve by building up triglyceride depots. Moreover, adipocytes and immune cells of the adipose tissue secrete adipokines, cytokines, lipokines and lipid mediators that function as paracrine and endocrine signals in the systemic metabolic regulation [1]. Adipose tissue is hence indispensable for a healthy metabolism and endocrine functioning. Excess development of adipose tissue, however, leads to obesity, which can trigger various chronic diseases, such as coronary heart disease, non-alcoholic fatty liver disease, renal and retinal vascular complications, insulin resistance and diabetes [2,3,4]. Worryingly, obesity-associated diseases affect more than 39% of the adult population today. Eventually, by 2060, the economic impact of obesity is projected to rise to 3.29% of the global gross domestic product [5].

Adipose tissue inflammation is a key factor leading to obesity-associated diseases. In response to excess fat storage, inflammasome activation and the pyroptosis of adipocytes [6,7,8], the infiltration of inflammatory immune cells and the release of danger signal lipid metabolites [9,10] ignite local inflammation in the fat depots [11]. Gene networks controlled by Toll like receptors 3 and 4 (TLR3 and TLR4, respectively) as well as by nuclear factor kappa B (NFκB) become eventually actively transcribed, resulting in the excessive production of pro-inflammatory cytokines and reactive oxygen species [12]. Adipose tissue inflammation impedes insulin sensitivity, may cause β-cell destruction in the pancreatic islets, and may also exacerbate autoimmunity [13,14].

Inhibiting adipose tissue inflammation hence has been long considered as a straightforward approach to abrogate obesity-associated diseases [15]. This working model hallmarks the “classical” era of the study of adipose tissue inflammation in the first decades of the 21st century [1,15,16,17,18].

**Figure 1:**
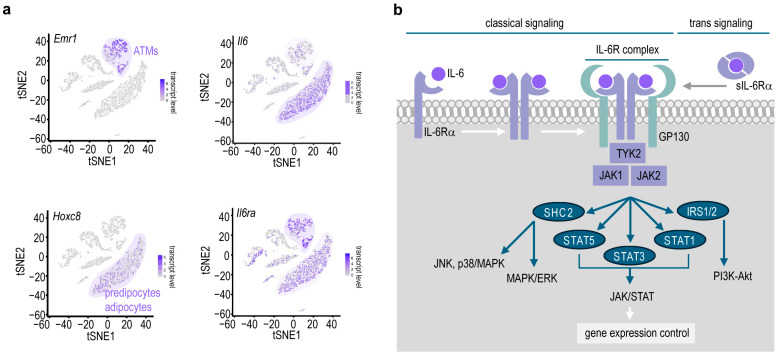
IL-6 signaling in the adipose tissue. (**a**) Single-cell sequencing data retrieved from Tabula Muris [35], showing cell populations of the mouse adipose tissue that express *Il6* and *Il6ra* (encoding IL-6 receptor α). *Emr1*: encodes F4/80 antigen, a marker protein of murine macrophages. Adipose tissue macrophages (ATMs) are F4/80-positive cells, and hence are identified as *Emr1*-expressing cells in this plot. Preadipocytes are identified as *Hoxc8*-expressing cells. *Hoxc8* encodes homeobox 8C, a protein marker of adipocyte precursors and adipocytes [36]. *Il6* expression is apparent in the preadipocyte/adipocyte population, and in a lesser extent, in the ATMs. In turn, *Il6ra* is strongly expressed by both ATMs and preadipocytes/adipocytes, allowing IL-6 to target both cell populations. tSNE: t-distributed stochastic neighbor embedding; purple color intensity of the cell populations is proportional to expression levels of *Emr1*, *Hoxc8*, *Il6* and *Il6ra* mRNA. (**b**) Simplified scheme of IL-6 signaling. In the so-called classical IL-6 signaling pathway IL-6 binds to IL-6 receptor α (IL-6Rα), which eventually forms dimers and associates with GP130 protein. The so-called trans-signaling is activated by soluble IL-6Rα (sIL-6Rα). SHC2: Src homology-2 containing protein; TYK: tyrosine kinase; JAK: Janus kinase; JNK: c-Jun terminal kinase; MAPK/ERK: mitogen-activated protein kinase/extracellular signal-regulated kinase; p38/MAPK: p38 mitogen-activated protein kinase; STAT1, 3 and 5: signal transducer and activator of transcription 1, 3 and 5; IRS1/2: insulin receptor substrate 1 and 2; PI3K-Akt: Phosphoinositide 3-kinase—Ak strain-transforming; IL-6R: IL-6 receptor complex containing IL-6, IL-6Rα and GP130 in a 2:2:2 ratio.

Recent findings challenge this paradigm, showing that inflammatory cytokines associated with innate immune signaling are essential for the proper differentiation of adipocytes in the early postnatal life [19,20,21]; inflammatory cells may play metabolically beneficial roles during the progression of obesity [22]; and certain pro-inflammatory signal mechanisms are unrelated to the worsening of obesity [23], or may be even protective from obesity-associated diseases [24]. Interleukin-6 (IL-6) is one of the immune signals that have controversial effects in adipose tissue physiology (Figure 1a). Excessive IL-6 is associated with metabolic impairment in obesity: when IL-6 is released in excess, it may promote adipose tissue inflammation [2,25], insulin resistance and diabetes [26]. However, mice lacking *Il6* expression develop obesity [27], and both endogenous and exogenous IL-6 has anti-obesity effects [28,29], since IL-6 increases lipolysis and heat generation from stored fat. Recent findings show that IL-6 sustains healthy fat metabolism after birth [30,31], and increases free fatty acid and leptin release from adipocytes [32]. IL-6 may activate signaling pathways through signal transducer and activator of transcription 3 (STAT3), and STAT3 is necessary for adipocyte differentiation (Figure 1b) [33] and is essential for the development of thermogenic adipocytes [34]. Given these novel findings on the role of adipocyte IL-6 signaling, here, we provide an overview on the role of IL-6 in fat catabolism through thermogenesis.

## 2. Thermogenic Activity of the Adipose Tissue

Thermogenic adipocytes generate heat by uncoupled mitochondrial oxidative phosphorylation. In brief, free fatty acids, released through the lipolysis of fat droplets, undergo β-oxidation in the adipocyte mitochondria, generating energy in the form of heat. Uncoupling protein-1 (UCP1), a transmembrane protein in the mitochondrial membrane, drives the re-entry of protons into the mitochondrial matrix and uncouples ATP synthesis from oxidative phosphorylation. The abundance of mitochondria combined with the expression of UCP1 evokes thermogenic potential in adipocytes, which eventually allows non-shivering thermogenesis, protects the core body temperature, and increases energy expenditure [37]. The loss of thermogenic adipocytes appears in aging and in obesity and is associated with worsened insulin sensitivity and glycemic control [38,39].

Thermogenic adipocytes may form brown adipose tissue depots, e.g., interscapular brown adipose tissue depot in rodents, or perirenal brown adipose tissue in human. Thermogenic adipocytes may also be scattered within fat-storing white adipose tissue depots [40]. In the latter case, the thermogenic adipocytes are often termed as beige or brite (brown in white) adipocytes. Dissipating nutritional energy as heat may help reduce body fat, and hence stimulating adipocyte thermogenesis—by so-called “adipose tissue browning”—may be a target of pharmacological interventions in obesity management [41].

## 3. Interleukin-6 Signaling in the Adipose Tissue

IL-6 is a glycoprotein encoded by the IL6 gene located in the 7p15.3 locus in human. Historically, IL-6 was described as interferon-β2, T-cell replacing factor, B-cell differentiation factor, reflecting to some of its effects [42]. The major stimuli of IL-6 production are pro-inflammatory signals, such as T-cell antigenic stimulation, TLR4-activating lipopolysaccharides, various viruses, interleukins, tumor necrosis factor beta, platelet-derived growth factor, protein kinase C, and elevated intracellular cyclic adenosine monophosphate (cAMP) levels [43,44]. IL-6 is abundantly produced in a pro-inflammatory tissue environment, such as in the obese adipose tissue. Accordingly, obese adipose tissue expresses IL6 mRNA and secretes IL-6 protein excessively [45,46,47,48].

Under physiological conditions, developing preadipocytes [49,50], and to a lesser extent, adipose tissue macrophages (ATMs) [21,51] are sources of IL-6 in the adipose tissue (Figure 1a). In obesity, ATMs are the most relevant source of IL-6 [51]. Interestingly, despite pro-inflammatory gene expression being a hallmark of adipocyte maturation [52], IL-6 expression is more prominent in adipocyte precursors than in mature adipocytes [49,50]. The association of increased adipose tissue IL-6 production with metabolic syndrome, increased blood glucose and insulin resistance has been confirmed in human and in various animal models of obesity [53,54,55].

IL-6 binds to IL-6 receptor subunit alpha (IL-6Rα), which forms tetramers with the GP130 co-receptor protein in the classic or trans-signaling pathways (Figure 1b), and eventually activates the Janus kinase 2 and signal transducer and activator of transcription 3 (JAK2/STAT3) signaling pathway [56,57,58]. In addition, IL-6 may also stimulate c-Jun terminal kinase, mitogen-activated protein kinase, extracellular signal-regulated kinase, p38, insulin receptor substrate 1 and 2, phosphoinositide 3-kinase, and SH2-containing protein tyrosine phosphatase-2/GRB2-associated-binding protein/mitogen-activated protein kinase (SHP2/Gab/MAPK) cascades (Figure 1b) [57,58]. IL-6RA is strongly expressed by ATM neutrophils, naïve CD4^+^ T-cells, preadipocytes and adipocytes (Figure 1a); therefore, cells of the adipose tissue can directly respond to IL-6 through the classical IL-6 signaling pathway [59].

## 4. The Role of IL-6 in Adipose Tissue Inflammation and Insulin Resistance

In the metabolically active organs, IL-6-mediated STAT3 activation destabilizes insulin receptor substrate 1 (IRS1) protein, causing insulin resistance. IRS1 is a signaling adapter protein with a key role in transmitting insulin receptor signals to intracellular pathways. IL-6 enhances the expression of suppressor of cytokine signaling protein 3, which causes phosphorylation and induces a conformational change in the functional domain of IRS-1; in this way, IL-6 impairs insulin action [60]. On the other hand, the inactivation of membrane-bound IL-6Rα signaling in hepatocytes and macrophages results in impaired insulin sensitivity [61]. Adipose tissue-derived inflammatory mediators may cause β-cell death in the pancreatic islets [62]. However, IL-6 appears to protect from pancreatic islet damage in obesity. IL-6 stimulates insulin secretion by β-cells in vitro and promotes the in vivo release of glucagon-like peptide 1 by α-cells, which may impede β-cell loss [63]. IL-6 directly impedes β-cell death through various mechanisms, involving autophagy, reduced cAMP levels and the reduced production of reactive oxygen species [63,64].

ATMs may be both sources and targets of IL-6. The ATM number is strongly increased in obese adipose tissue, concomitant with the pro-inflammatory activation of ATMs and with their IL-6 synthesis [11]. However, IL-6 may also induce a pro-resolving macrophage activation through the JAK2/STAT3 signaling pathway [62] and through CCAAT/enhancer-binding protein beta (C/EBPβ) expression [65]. The underlying mechanism involves the upregulation of IL-4 receptor in ATMs, allowing them to respond to IL-4, a relevant anti-inflammatory signal [66,67,68]. IL-10, which is a pro-resolving cytokine, can also increase IL-6 expression in macrophages [69]. It is plausible that IL-6 has an overlapping effect with IL-10 and synergizes with IL-4 to determine macrophage phenotype [68]. Moreover, IL-6 may increase the number of pro-resolving ATMs, at least in part due to an increased local proliferation of ATMs [66]. The local proliferation of ATMs may be metabolically harmful; however, when this is associated with a pro-resolving activation, expansion of the pro-resolving ATM pool may protect from metabolic inflammation [70].

IL-6 is a proliferative stimulus for a T helper cell lineage [71,72,73], termed as Th17 cells, since the main cytokine produced by these cells is IL-17. The function of Th17 cells is host defense at barrier tissues including gut, skin, and lung. Th17 lymphocytes can be an important link between obesity and adipose tissue inflammation. An increased number of Th17 lymphocytes has been found in the adipose tissue and in the blood in obese animal models [74,75]. In children with obesity and diabetes mellitus, an increased Th17 cell number was found in the peripheral blood [76]. Another study supported this finding from a different perspective: weight reduction in children led to a significant decrease in peripheral Th17 cells. Obesity also enhances the expansion of an IL-6-positive natural killer (NK) cell subpopulation. These NK cells trigger metabolic inflammation, induce pro-inflammatory ATM activation, and aggravate insulin resistance [61]. This metabolically harmful NK population expands in obesity and secretes tumor necrosis factor alpha (TNF*α*), which ignites low-grade inflammation, increases plasma IL-6 levels, and impairs insulin sensitivity [60].

## 5. Hypothalamic IL-6 Signaling May Protect from Obesity

**Figure 2:**
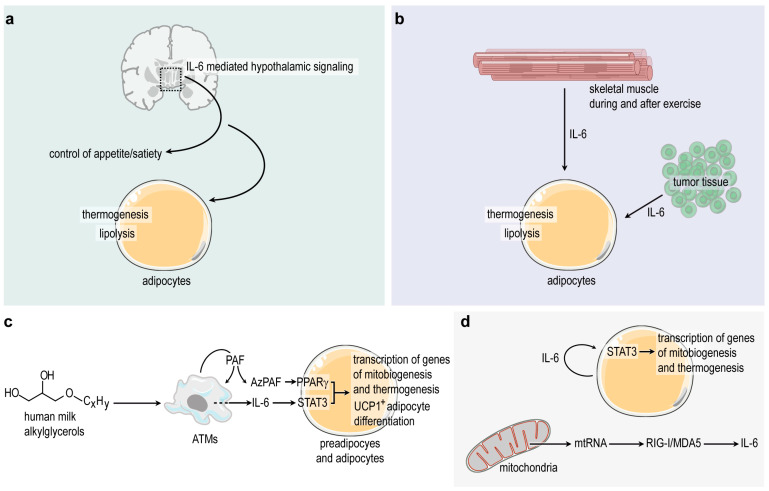
Possible effects of IL-6 signaling on energy intake and energy expenditure under non-obesogenic conditions. (**a**) Hypothalamic IL-6/STAT3 signaling may reduce energy intake and stimulate thermogenesis and lipolysis in adipocytes. The underlying mechanisms are still largely unexplored. (**b**) IL-6 is released from the skeletal muscle during exercise, which may increase lipolysis and thermogenesis in adipocytes. Tumor tissues may also secrete IL-6 into the blood stream, that can over-stimulate fat catabolism and cause excessive wasting of body fat and lead to cachexia. (**c**) It has been shown recently that IL-6/STAT3 signaling triggers the expression of genes required for mitochondrial biogenesis and thermogenesis in preadipocytes and adipocytes, and STAT3 is necessary for thermogenic fat development. Upstream stimulators of the locally produced IL-6 may be human milk alkylglycerols (AKGs). AKGs are metabolized by ATMs into platelet-activating factor (PAF), which is non-enzymatically converted into azelaoyl PAF (AzPAF) and stimulates IL-6 release from ATMs. AzPAF is a peroxisome proliferator-activated receptor gamma (PPARγ) ligand [21]. (**d**) Autocrine IL-6 signaling stimulates the transcription of mitobiogenesis and thermogenesis genes in adipocytes, especially during the early postnatal life. Upstream stimulators of IL-6 synthesis are mitochondria-derived RNA species (mtRNA), which increase IL-6 synthesis through the RIG-I/MDA5 (retinoic acid-inducible gene-1/RIG-I-like receptor dsRNA helicase) cytosolic RNA recognition pathway [31].

Obesity is associated with an increased IL-6 serum level [77], while there is a reduced IL-6 level in the central nervous system (CNS) in overweight and obese patients [78]. Central IL-6Rα activation and IL-6 action is enhanced in obesity [78]. IL-6 is produced in both neuronal and glial cells [79] in the CNS. IL-6-activated STAT3 signaling in hypothalamic neurons suppresses food intake and improves peripheral glucose homeostasis [61], although the underlying molecular mechanisms are still unexplored (Figure 2a). Glucagon-like peptide-1 (GLP-1) acts as an anorexigenic hormone in the lateral hypothalamus to reduce food intake. IL-6 is an important downstream mediator of the weight loss effects induced by central glucagon-like peptide-1 receptor (GLP-1R) stimulation [80]. Recent studies have shown that IL-6 and leptin share the same molecular effector pathways to control energy and glucose homeostasis through the activation of STAT3, which eventually controls the release of anorexigenic and orexigenic neuropeptides [19]. IL-6 also acts on the parabrachial nucleus to reduce food intake and to increase brown adipose tissue thermogenesis by increasing the hypothalamus–pituitary gland–thyroid axis activity and sympathetic nerve activation. A reduction in IL-6 in parabrachial nucleus increases body weight and adiposity, reduces adipose tissue thermogenesis, and increases food intake. After IL-6 loss in the parabrachial nucleus, the thermogenic “brown” adipose tissue depot of mice has a decreased expression of genes necessary for thermogenic fat development and functioning [81], suggesting that hypothalamic IL-6 signaling stimulates thermogenesis in the adipose tissue and eventually may increase energy expenditure (Figure 2a).

Moreover, the infusion of IL-6 increases systemic lipolysis and fatty acid oxidation in human [82] and inhibits lipogenesis in adipocytes in vitro [83]. Similarly, physical exercise triggers a transient increase in blood IL-6 levels [84], and this skeletal muscle-derived IL-6 might help fat catabolism in adipocytes (Figure 2b). The concentration of *IL6* mRNA in monocytes does not increase following physical activity; therefore, immune cells are unlikely to be responsible for the elevated plasma IL-6 levels observed during and after exercise [85]. Recent studies have showed that IL-6 is produced in the skeletal muscle during contractions of Type 2 muscle fibers. IL-6 production is especially high when glycogen levels become critically low. It is hypothesized that there is an IL-6-mediated signaling to the liver that stimulates hepatic glucose production and supports muscle contractions [86]. Also, muscle glucose uptake is impaired in IL-6-knockout (IL-6-KO) mice and exercise training fails to improve diet-induced insulin resistance in IL-6-KO mice [87].

## 6. Local IL-6 Signaling Stimulates Thermogenic Fat Development through STAT3

Thermogenic fat depots—often termed as brown, beige or brite fat depots—are the sites of dissipating nutritional energy as heat via non-shivering thermogenesis. In brief, thermogenic fat depots may use stored fat for heat production through uncoupled oxidative phosphorylation [80]. As a first step, free fatty acids are released by lipolysis from fat droplets and are eventually catabolized in β-oxidation in the mitochondria. Thermogenic adipocytes express UCP1 [37] that uncouples oxidative phosphorylation from ATP synthesis, releasing heat. UCP1 is a transmembrane protein located in the mitochondrial inner membrane, and it drives the re-entry of protons to the mitochondrial matrix during ATP synthesis. Thermogenic adipocytes have large number of mitochondria, express UCP1 and store fat in multilocular lipid droplets: all these traits maximize the capacity of breaking down lipids and generate heat from fat [88]. This mechanism allows non-shivering thermogenesis. Since shivering thermogenesis depends on muscle mass, heat production in non-shivering thermogenesis is a task of the newborn adipose tissue, and when this is compromised, excessive fat accumulation—obesity—may occur [89].

**Figure 3:**
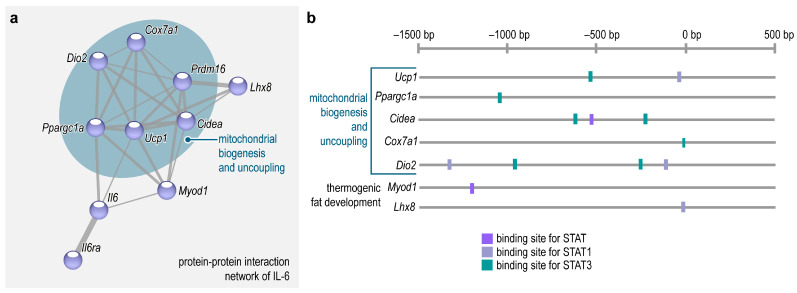
IL-6/STAT3 stimulates expression of genes necessary for mitochondrial biogenesis and thermogenesis. (**a**) STRING protein–protein interaction network of IL-6 [91]. A gene network required for mitochondrial biogenesis and uncoupling is strongly associated with IL-6 signaling. The gene network includes *Ucp1*, encoding uncoupling protein 1; *Ppargc1a*, encoding peroxisome proliferator activated receptor gamma coactivator 1; *Cidea*, encoding cell death-inducing DFFA-like effector A; *Prdm16*, encoding PR domain containing 16; *Dio2*, encoding Type II iodothyronine deiodinase; and *Cox7a1*, encoding cytochrome c oxidase subunit 7A1. Moreover, *Lhx8* and *Myod1*, which encode proteins (LIM homeobox 8 and myogenic differentiation 1, respectively) involved in thermogenic adipocyte development, are also associated with the gene network [39,92]. (**b**) Scheme summarizing transcription binding sites upstream to promoter regions of *Ucp1*, *Ppargc1a*, *Cidea*, *Cox7a1*, *Dio2*, *Myod1* and *Lhx8*. All these genes have predicted binding sites for STATs, STAT1 and STAT3. Transcription factor binding sites were predicted by Interferome 2.0 [93].

Key genes necessary for mitochondrial biogenesis—i.e., the expansion of the mitochondrial network, allowing increased β-oxidation and mitochondrial thermogenesis—as well as genes of thermogenic adipocyte development and mitochondrial uncoupling form a gene network associated with IL-6 signaling (Figure 3a). Coherently, several genes of mitochondrial biogenesis and uncoupling have binding sites for STAT3 in their promoter regions (Figure 3b), suggesting that IL-6 may control the expression of these genes [90]. Indeed, IL-6 produced within the adipose tissue appears to stimulate thermogenic fat differentiation by autocrine and paracrine mechanisms [21,31,50] (Figure 2c,d).

Breastfeeding maintains thermogenic adipocyte differentiation and might reduce the risk of having obesity during early childhood [30,94]. Human milk-specific alkylglycerol-type ether lipids (AKGs) maintain thermogenic adipose tissue in young mice (Figure 2c). The underlying mechanism requires ATMs, which colonize the developing adipose tissue at birth [21,95,96] and metabolize nutritional AKGs to platelet-activating factor (PAF). PAF functions as a paracrine signal to preadipocytes and adipocytes and activates an autocrine IL-6/STAT3 signaling (Figure 2c). Eventually, IL-6 stimulates the expression of genes associated with mitochondrial biogenesis and thermogenesis in mouse and human adipocytes [21,31,50], and this effect is dependent on the expression level of IL-6 receptors in the fat depot [97]. Coherently, IL-6-deficient mice have a decreased ability to develop thermogenic fat depots in response to cold exposure [98,99], IL-6 treatment increase thermogenic fat mass in mice [100], the UCP1^+^ fat expresses higher level of PAF receptors than its UCP1^−^ counterpart in mouse [31], and mice lacking PAF receptor expression lose UCP1^+^ adipocytes [21].

Similarly, there is an autocrine IL-6 stimulation of the expression of genes required for mitochondrial biogenesis and thermogenesis in young adipocytes [89] (Figure 2d). Upstream signals for this endogenous IL-6 synthesis are double-stranded mitochondrial RNA species, which are released from the mitochondria [89]. In this scenario, mitochondrial RNA acts as a signal to stimulate the nuclear expression of genes required for mitochondrial assembly and heat production (Figure 2d).

The initiation of labor—parturition—is associated with pro-inflammatory signals. The activation of TLR- and NFκB-controlled gene expression leads to the production of pro-inflammatory cytokines, including IL-6, that trigger a sequence of physiological events leading to delivery [101]. Concomitant to this inflammatory burst, there is a rapid metabolic reprogramming of the fat depots at birth, to provide sufficient energy for heat production and ATP synthesis. It appears that inflammatory signals—at least in part, the level of IL-6—determine whether adipocytes accumulate fat or generate heat and ATP in the early postnatal life [31,102]. Similarly, IL-6 signaling along with a type I interferon (IFN-I) response is essential for heat production from fat in the early postnatal life [21,31,103].

In adulthood, the stimulation of β-adrenergic receptors, the key signal for adipocyte lipolysis and thermogenesis, triggers IL-6 synthesis in mouse and human adipocytes, that, at least in part, may mediate the effects of noradrenaline on adipocytes [46].

## 7. IL-6-Induced Fat Loss and Cachexia

Cachexia is a pathological wasting syndrome characterized by muscle, and adipose tissue loss which cannot be reversed by nutrition [104]. It is well documented in cases of chronic obstructive pulmonary disease, congestive heart failure, rheumatoid arthritis, chronic kidney disease, AIDS, and cancer. Wasting in cancer cachexia was initially thought to be caused by the energy requirements of the tumor itself; however, the size and energy uptake of the tumor does not correlate with the degree of cachexia [105]. Several so-called “cachexokines”—cachexia-associated cytokines—have already been identified, including IL-6 (Figure 2b) [106].

The expression of lipolytic genes is increased in the adipose tissue from patients with cancer cachexia. Activities of adipose triglyceride lipase and hormone-sensitive lipase are also increased in cachexia. In addition to lipolytic genes, there is an increased expression of genes associated with thermogenesis in cachectic adipose tissue. It is plausible that an increase in thermogenic competence—so-called adipose tissue “browning”—induces an increase in energy expenditure that eventually contributes to cachexia [107]. IL-6 released from the tumor may be a direct inducer of lipolysis and thermogenesis in adipocytes [37,108,109]. For instance, cell death-inducing DNA fragmentation factor-α-like effector A (CIDEA) protein is associated with lipolysis [110] and thermogenesis [105], and its expression is stimulated by IL-6/STAT3 signaling [21] (Figure 3a,b). Human CIDEA is located on the surface of lipid droplets and its expression is increased in the adipose tissue of cachectic cancer patients. The CIDEA level correlates positively with plasma levels of free fatty acids—indicators of lipolysis—and weight loss [111]. A lack of energy triggers the adenosine monophosphate-activated protein kinase (AMPK), which is a cellular energy sensor. Active AMPK promotes energy conservation by activating catabolic pathways and inhibiting anabolic pathways. The CIDEA-mediated degradation of AMPK is essential for tumor-induced lipolysis [112]; hence, IL-6-induced CIDEA expression may explain the excessive fat catabolism in cachexia [111].

The blood level of IL-6 correlates with the amount of weight loss in cancer patients, and an elevated IL-6 level shows a positive correlation with reduced patient survival [16]. Moreover, neutralizing IL-6 receptors with an antibody in cachectic mice inhibits lipolysis and protects from excessive fat loss [48]. A systematic review of studies involving 1537 patients receiving IL-6-blocking therapy (Tocilizumab treatment) shows that IL-6 blocking causes significant weight gain, providing further evidence for a role of IL-6 in body weight regulation [113].

## 8. Conclusions and Outlook

**Figure 4:**
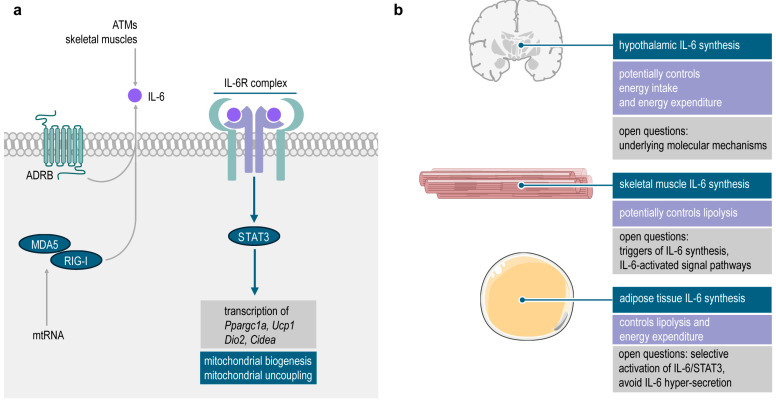
Summary of the possible role of IL-6 in thermogenesis in adipocytes. (**a**) IL-6 may be released by contracting skeletal muscle fibers and reach the adipose tissue through the bloodstream or may be released locally by ATMs and by further immune cells of the adipose tissue. IL-6 may also be produced by preadipocytes or adipocytes in response to β-adrenergic receptor (ADRB) stimulation, or cytosolic mitochondrial RNA (mtRNA), allowing autocrine IL-6 signaling. Activation of the IL-6/STAT3 pathway stimulates the transcription of genes required for mitochondrial biogenesis and thermogenesis. (**b**) At a systemic level, IL-6 signaling may affect adipocyte thermogenesis by various mechanisms: it may affect the hypothalamic control of energy intake and energy expenditure. Skeletal-muscle-derived IL-6 may stimulate thermogenesis in adipocytes. Adipose tissue-derived IL-6 may stimulate lipolysis and thermogenesis. All these mechanisms have the potential to increase fat loss; however, the underlying molecular mechanisms are still to be explored.

Since the initial reports on the metabolically harmful effects of IL-6, several lines of study have shown that IL-6 is a physiological signal that regulates thermogenic adipose tissue development and fat catabolism (Figure 4a). From a clinical perspective, stimulating IL-6/STAT3 signaling in the adipose tissue may augment adipose tissue browning and increase fat loss. Its therapeutic applications may include an improved body weight control by increasing fat catabolism. In turn, abrogating IL-6 signaling in the cachectic adipose tissue may overcome pathological fat loss.

Macrophage-, and adipocyte-derived IL-6 triggers the transcription of genes required for mitochondrial biogenesis and thermogenesis (Figure 4a). In the newborn infant, nutritional exposure to human milk-specific lipids appear to stimulate a local IL-6 synthesis, that favors the development of thermogenic adipocytes. Eventually, IL-6 signaling in the developing adipose tissue may protect from excess adiposity. In adults, hypothalamic IL-6 signaling contributes to the central regulation of appetite. Furthermore, noradrenaline-stimulated IL-6 synthesis in adipocytes, and skeletal muscle-produced IL-6 may enhance lipolysis and gluconeogenesis and reduce insulin resistance (Figure 4b). Local IL-6 synthesis in the adipose tissue may similarly induce fat catabolism (Figure 4b). While these effects of IL-6 may be metabolically beneficial, excessive IL-6 synthesis may hyper-activate the thermogenic adipocyte differentiation and cause a pathological loss of fat (Figure 4b). Manipulating such a versatile cytokine for therapeutic purposes requires a nuanced understanding of its cell autonomous functions. Nevertheless, IL-6 appears as a powerful inducer of fat loss, and hence, regulation of adipose tissue IL-6 synthesis may emerge as a novel therapeutic target in obesity management.

In addition to the stimulation of IL-6 synthesis, targeting the downstream IL-6 signaling pathway may also be a straightforward approach to increase weight loss in obesity. However, little is known about the signal pathways activated by IL-6 in the adipose tissue, and to date our understanding is limited to the IL-6/STAT3 signal transduction in the context of the lipid metabolism of adipocytes. Indeed, we do not fully understand the mechanisms behind the hypothalamic effects of IL-6, and the signals that induce skeletal muscle, or adipocyte IL-6 release. A targeted activation of IL-6 signaling may help burning off energy stored in fat as heat, and in turn, inhibiting cachexia-associated IL-6 synthesis may help to block pathological weight loss and improve life quality.

In summary, IL-6 signaling may be a physiologically relevant although yet under-appreciated mechanism in body weight control and may be exploited by inducing adipose tissue browning to increase energy expenditure.

## Data Availability

Not applicable.
